# Adherence to antiretroviral therapy among HIV infected children measured by caretaker report, medication return, and drug level in Dar Es Salaam, Tanzania

**DOI:** 10.1186/1471-2431-13-95

**Published:** 2013-06-15

**Authors:** Frida William Mghamba, Omary MS Minzi, Augustine Massawe, Philip Sasi

**Affiliations:** 1 Department of Paediatrics and Child Health, School of Medicine, Muhimbili University of Health and Allied Sciences, P.O. BOX 65001, Dar-Es-Salaam, Tanzania; 2 Unit of Pharmacology and Therapeutics, School of Pharmacy, Muhimbili University of Health and Allied Sciences, P.O. BOX 65013, Dar Es Salaam, Tanzania; 3 Department of Clinical Pharmacology, School of Medicine, Muhimbili University of Health and Allied Sciences, P.O. BOX 65010, Dar-Es-Salaam, Tanzania

## Abstract

**Background:**

Adherence to antiretroviral drugs in the treatment of paediatric HIV infection is complicated because of many factors including stigma and drug intake logistics. It is therefore important to identify children with non-adherence in order to intervene before they become at risk of developing treatment failure or drug resistance. The aim of this study was to determine the level of adherence to antiretroviral therapy (ART), measured by caretaker report, medication return and nevirapine plasma concentration. In addition, the association between level of adherence and patient’s immune status was compared across the three methods of measuring adherence.

**Methods:**

This was a descriptive cross-sectional study involving HIV infected children aged 2–14 years, on nevirapine- based antiretroviral treatment for at least six months, attending care and treatment clinic in three municipal hospitals in Dar- Es- Salaam City. Eligible patients and their accompanying caretakers were consecutively enrolled after obtaining written informed consent. Structured questionnaires were administered to caretakers to assess patient’s adherence by caretaker report and medication return whereas a single blood sample for CD4 cell count/percent and determination of nevirapine plasma concentration was taken from patients on the day of assessment.

**Results:**

A total of 300 patients and accompanying caretakers were enrolled and the mean patient age (SD) was 8 (3) years. Caretakers’ report and medication return showed good adherence (98% and 97%) respectively. However, the level of adherence assessed by nevirapine plasma concentration (85%) was significantly lower than caretaker report and medication return (p < 0.001). The agreement between nevirapine plasma concentration and medication return and between nevirapine plasma concentration and caretaker report was weak (k = 0. 131) (k = 0. 09) respectively. Nevirapine plasma concentration below 3 μg/ml was associated with immunosuppression (p = 0. 021) whereas medication return (>5% of prescribed doses) and caretaker reported missing more than one dose within 72 hours prior to interview were not associated with immunosuppression (p = 0. 474), (p = 0. 569) respectively.

**Conclusion:**

Lower adherence level observed using nevirapine plasma concentration and its association with immunological response supports the validity of the method and indicates that adherence data obtained from caretaker report and medication return may overestimate the true adherence in paediatric antiretroviral therapy.

## Background

Introduction of antiretroviral therapy has resulted in a decrease in mortality and morbidity in HIV/AIDS patients worldwide [1]. Antiretroviral therapy (ART) suppresses viral load and raises the number of CD4 cells and thus the quality of life of HIV/AIDS patients on ART is improved. However, the efficacy of antiretroviral therapy depends on a high level of adherence [1,2]. Studies have shown that, for a successful ART, adherence to dosage regimen should be ≥ 95% [3]. Non-adherence to ART may lead to suboptimal drug levels, which may result in therapeutic failure, deterioration of the immune system and/or emergence of drug-resistant HIV strains [3,4].

Adherence to antiretroviral therapy in paediatric and adolescent patients has been reported to be problematic due to multiple factors. These include the pill burden, poor palatability, unpleasant flavour, side effects and long term toxicity [5]. In addition, caregiver type, income, disclosure of child, caregiver-child communication, caregiver health belief, depression, stress, stigma, children refuse to take medications and forgetfulness have also been associated with non-adherence [6,7]. Caretaker being too busy or frequently away from home and many other logistical challenges are contributing factors of low adherence to ART in paediatric patients [8].

In low- and middle- income countries, lack of a simple and cost-effective gold standard for measuring adherence to ART is a major challenge for paediatric HIV/AIDS treatment. A number of strategies such as pill count, electronic monitors, diaries and self-report questionnaires have been employed. However, each of these methods has limitations and can provide different estimates of adherence. Importantly, these limitations have been reported to be more pronounced in paediatric patients [9,10].

Despite its limitations, antiretroviral drug plasma concentration may be considered the gold standard. However, it is often not feasible, particularly, in resource-poor countries. In adult patients, the antiretroviral plasma drug concentration has been reported to correlate with viral load response; therefore, giving a more reliable adherence estimate than self reporting [11]. However, to the best of our knowledge, the literature on the use of plasma antiretroviral drug concentration to assess adherence in paediatric patients is limited.

Although studies done to establish an association between adherence (measured by self report and medication return) and immunological response among paediatric HIV/AIDS patients have shown conflicting findings [12-14], adherence is a good predictor of effective virological suppression and subsequent immunological recovery. Some studies have shown and others have not shown an association between adherence by self report or medication return and immunological response. Apart from non-adherence, immunological suppression has been found to correlate with severity of malnutrition, advanced stage of HIV disease (WHO classification) and increase likelihood of opportunistic infection [15,16]. Irrespective of the method of measurement, adherence to antiretroviral treatment is critical in order to reduce paediatric HIV/AIDS related mortality. We hereby report the level adherence to antiretroviral therapy and its association with immune status among paediatric HIV/AIDS patients in Dar-Es-Salam.

## Methods

This was a descriptive cross-sectional study conducted from May to October 2011 at three municipal hospitals (Mwananyamala, Temeke and Amana) in Dar-Es Salaam, Tanzania. HIV/AIDS patients aged 2-14 years on paediatrics nevirapine-based antiretroviral treatment regimen, for at least six months, and their accompanying caretakers were enrolled consecutively as they attended HIV care and treatment clinic. The study protocol was reviewed and approved by the Muhimbili University of Health and Allied Sciences (MUHAS) Ethical Review Committee and permission to conduct the study was obtained from the respective District Medical Officers. Only eligible patients whose accompanying caretaker gave a written informed consent were enrolled and patients not on nevirapine-based regimen were excluded. Also excluded were patients on medications that are known to induce/inhibit nevirapine metabolizing enzymes (CYP 2B6 and CYP3A4) such as rifampicin, fluconazole, and ketaconazole. An assent was also obtained from patients aged 12 years and above before inclusion into the study.

### Study procedure

A structured questionnaire was used to assess adherence to antiretroviral therapy and information on the patient’s concurrent use of other drugs and co-morbidity was collected. In addition, patient’s medical history including history of illness in the previous visit, history of cough, fever, ear discharge, vomiting of medication, skin or oral lesion was taken. The duration of antiretroviral therapy, the time at which the early morning dose prior to assessment was taken, and the type of ART regimen in use were checked and recorded. Socio-demographic characteristics of the caretaker were also recorded. Screened patients in the study were on ART regimens in line with the Tanzania guideline for diagnosis and management of HIV infection. All patients were on Paediatric Fixed Dose Combination (FDC) tablets, and patients who were selected into the study, were either on: Triomune Baby, FDC 6® (50 mg nevirapine, 6 mg stavudine, 30 mg lamivudine) or Triomune Junior, FDC 12® (100 mg nevirapine, 12 mg stavudine, 60 mg lamivudine) or a fixed-dose combination containing 60 mg Zidovudine, 30 mg lamivudine, and 50 mg nevirapine. The nevirapine dose was 160 - 200 mg per m^2^ per dose given twice a day [17]. However, the fixed-dose combination tablets were administered according to body weight as recommended by WHO. Thus children weighing 12–13.9 kg received 200 mg; 13–19.9 kg received 250 mg; 19.9 - 24.9 kg received 300 mg and those weighing ≥25 kg received 400 mg of nevirapine per day in two divided doses. A detailed physical examination was then performed by the attending clinician and this included body weight, height and mid upper circumference measurement.

A single blood sample for determination of nevirapine plasma concentration and CD4 cell count/percentage was collected and divided into two aliquots. The aliquot for CD4 cell count/percentage was placed in Ethylenediaminetetraacetic Acid (EDTA) tubes and transported to the Muhimbili HIV Reference Laboratory for flow cytometry whereas the aliquot for nevirapine plasma concentration was placed in heparinised tubes and immediately centrifuged to obtain plasma. The plasma samples were transferred into cryovials, transported in a cool box to the MUHAS-Sida Bioanalytical Laboratory and stored at −80°C until assay. Determination of nevirapine plasma concentration was done using a published reversed phase High Performance Liquid Chromatography (HPLC) method [18]. Before analysis of patient samples, the method was validated with respect to accuracy and precision. The method was also tested for lack of interference of endogenous substances and the components of cotrimoxazole (sulphamethoxazole and trimethoprim) since most patients are given cotrimoxazole before or during ART.

### Assessment of adherence

Adherence to antiretroviral therapy was assessed using caretaker report, medication return and nevirapine plasma concentration. The caretaker reported the total number of missed doses for the past three days. Adherence was concluded when no more than one dose was missed three days prior interview. A patient assessed by medication return was categorized as adherent when less than 5% of the medications were returned on the day for a refill and the level of adherence was calculated as percentage of prescribed dose not returned. Nevirapine trough plasma concentration of 3 μg/ml has been shown to be the cut off for efficacy [19]. Therefore, nevirapine plasma concentration ≥ 3 μg/ml was categorized as adherence. Adherence obtained by nevirapine plasma concentration as an objective method was compared with subjective methods, care-taker’s report and medication return.

### Sample size

The sample size was calculated based on the comparison of two independent proportions from groups of unequal sample size (good immune status versus immunosupressed). Since no similar previous study could be traced in the literature, the proportion of patients with good immune status (π_1_) was assumed to be 50% and the proportion of immunosupressed children (π_0)_ to be 30%. Taking 5% significance level, and an 80% power to detect a significant difference, the minimum sample size was calculated to be 200 and thus a total of 300 patients (100 from each hospital) was recruited into the study.

### Predictor variables

The predictor for immune status in this study were adherence and non-adherence measured by caretaker report, medication return and nevirapine plasma concentration.

### Possible confounders

The possible factors that could affect adherence to ART and immune status in this study were: demographic factors of caretaker (Age, sex, occupation, marital status, level of education, religion); age and sex of the patient; caretaker type; disclosure of child; and duration of ART**.** Others included clinical factors such as infections (e.g. pneumonia, diarrhoea, malaria, otitis media, oral lesion, stage of HIV disease and nutritional status.

### Outcome variables

In this study, immune status was defined and graded according to WHO immunological classification [17]. Good immunity was defined as the CD4 cell % >30% for patients under 5 years or CD4 cell count >500/μl for patients above 5 years. CD4 cell % or count below the ones quoted above was defined as immunosuppression.

### Statistical analysis

Data were entered in the Epi info software version 3.5.1 and then exported to the Statistical Package for Social Science (SPSS) version 15 for analysis. Mean and standard deviations were calculated for continuous variables whereas percentages were calculated for categorical variables. All continuous variables were categorized and analyzed using the Chi square test or Fisher’s exact test and P value < 0.05 was considered significant. The association was measured by Odds ratio (OR). Factors with P-value 0.2 or less at bivariate analysis were selected for further multivariate analysis and entered into a logistic regression model. The model was used to assess the independence and strength of association of predictive factors of interest using CD4 count as the outcome variable, as well as to control for possible confounders of the main predictor (adherence) and outcome. A difference of at least 10% between adjusted odds ratio and the crude odds ratio was considered confounding. The Kappa statistic was used to assess agreement between adherence measures (caretaker report, medication return and nevirapine plasma concentration). The sensitivity, specificity, positive predictive value and negative predictive value were used to determine the validity and precision, using CD4 cell count/percentage, to compare the three methods of measuring adherence (caretaker report, medication return and plasma drug concentration) to detect adherence and non adherence.

## Results

### Study population

A total of 300 participants met inclusion criteria and were recruited into the study. The majority of these patients were above five years (81.3%) of age. The mean age (SD) was 8 (3) years and almost half of the participants were female (50.7%).

The majority (77%, n = 231) had advanced stage of HIV disease and almost half were malnourished. Of the 176 children assessed for disclosure only 23.9%, (n = 42) with age of 7–14 years knew their HIV status. Most of the children (90.7%, n = 272) were on ART for more than one year and 31.7% (n = 95) had infections. More than three quarters (77.7%, n = 233) of children were immunosuppressed at baseline while only 28% (n = 84) were immunosuppressed when current CD4 cell %/ count was checked.

### Socio-demographic characteristics of caretakers

The mean age of the caretakers was 35.9 ± 10.7 years ranging from 18 to 70 years. Of the 300 caretakers, 88.3% (n = 265) were female and only 21% (n = 63) had beyond primary school education. Most of the caretakers had primary school education (68.7%, n = 206). The majority of caretakers (58.7%, n = 176) were mothers of patients. Among the 300 caretakers 36% (n = 138) were married and only 16.7% (n = 50) were employed**.**

### Level of adherence

**Figure 1:**
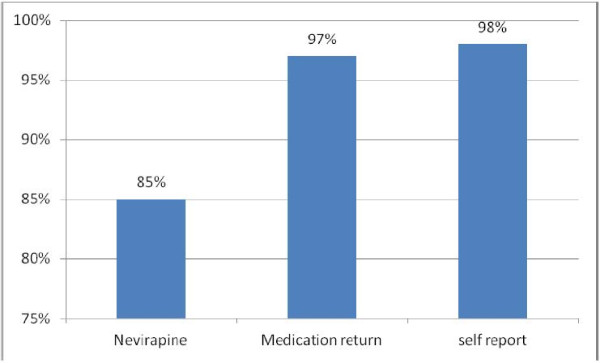
Proportion comparison of adherence.

As many as 85% of the participants (n = 254) were adherent when assessed by nevirapine concentration whereas the proportion of adherence assessed by medication returned and caretaker report were (97%, n = 291), (98%, n = 295) respectively (Table 1). The level of adherence assessed by nevirapine plasma concentration was significantly lower (85%) than that suggested by medication return and caretaker report (both p < 0.01). However, there was no significant difference in the level of adherence by caretaker report and medication return (p = 0. 06) (Figure 1). Agreement between nevirapine plasma concentration and caretaker report and between nevirapine plasma concentration and medication return was weak (k = 0. 01) (p = 0.77), (k = 0.13) (p = 0.002) respectively (Table 2).

**Table 1:** Level of adherence to ARV among HIV children

**Variable**	**Frequency (N)**	**Proportion**	95% CI*
**1. Medication return**			
≤5%	291	0.97	
>5%	9	0.03	0.02-0.06
**2. Caretaker self report**			
Never missed	295	0.98	
Missed	5	0.02	0.01-0.03
**3. Nevirapine conc**			
≥3 μg/ml	254	0.85	
<3 μg/ml	46	0.15	0.12-0.20

* Confidence interval.

**Table 2:** Agreement between nevirapine plasma concentration versus medication return and caretaker report

**Variables**	**Nevirapine plasma concentration <3 μg/ml**	**Nevirapine plasma concentration ≥3 μg/ml**	**Kappa value**	**P-value**
			( 95%CI*)	
**Medication return**				
>5%	5	5	0.13(0.007-0.011)	0.002
≤5%	41	249		
**Caretaker report**				
Missed	1	4	0.01(1–1)	0.77
Not missed	45	250		

*Confidence interval.

### Adherence measures and immune status

Nevirapine plasma concentration < 3 μg/ml was associated with immunosuppression (OR = 2.84, p = 0.002) whereas adherence by caretaker report as well as by medication report were not associated with immunosuppression (OR = 0.64, p = 0.569) and (OR =1.75, p = 0.474) respectively (Table 3). Compared with immune status (CD4 count/percentage), the sensitivity of nevirapine plasma concentration for detecting inadequate adherence was higher (11.1%) compared to medication return and caretaker report (4.7% and 1.2% respectively).

**Table 3:** Bivariate analysis of adherence measures and immune status (N = 300)

**Immune status**					
**Variable**	**Immunosuppression**	**Normal**	**OR**	**CI**	**P Value**
	**n (%)**	**n (%)**			
**1. Caretaker report**					
Missed	1(20.0)	4(80.0)	0.64	0.07-5.79	0.569
Not missed	83(28.1)	212(71.9)	1		
**2. Medication return**					
>5%	4(40.0)	6(60.0)	1.75	0.48-6.36	0.474
≤5%	80(27.6)	210(72.4)	1		
**3. Niverapine plasma concentration**					
<3 μg/ml	22(47.8)	24(52.2)	2.84	1.49-5.41	0.002
≥3 μg/ml	62(24.4)	192(75.6)	1		

### Other factors associated with immune status

Other factors associated with immunosuppression were the age of the patient ,2-5 years (OR = 5.52, CI 2.8-10.6) , patients on antiretroviral therapy for less than one year (OR = 6.27, CI 2.69-14.63) and infections (OR = 2.00, 1.18-3.37). These infections included pneumonia, Otitis media, oral lesion and skin lesion.

### Multivariate analysis of the factors associated with immune status

The final multivariate analysis was performed by a backward elimination procedure taking into account the biological knowledge about independent variables and how they relate to immune status. After controlling for confounders, nevirapine plasma concentration <3 μg/ml was independently associated with immunosuppression (OR = 2.47, CI = 1.15–5.33). Duration of antiretroviral therapy (ART) less than a year was also independently associated with immunosuppression (OR = 6.79. CI = 2. 69–17.16). The potential confounders of nevirapine plasma concentration were patients with young age (2-5 years) (OR = 6.00, CI =2.96–12.17) and infections (OR = 2.00, CI = 1.01–3.41) (Table 4).

**Table 4:** Multivariate analysis of the factors associated with immune status (N = 300)

**Variables**	OR (95%CI*)	**P-value**
**Nevirapine plasma conc**		
<3 μg/ml	2.47(1.15-5.33)	0.021
≥3 μg/ml		
**Age of the child**		
2-5 years	6.00(2.96-12.17)	0.0001
6-8	5.80(2.60-12.94)	0.0001
9-14	1	
**Overall Infections**		
Yes	2.00(1.01-3.41)	0.05
No	1	
**Duration of ARV**		
≤1 year	6.79(2.69-17.16)	0.0001
>1 year	1	

OR- Odd Ratio * Confidence interval.

## Discussion

In this study, we have estimated the level of adherence to ART in paediatric HIV/AIDS patients using two commonly employed methods (caretaker report, and medication return) and one seldom used method (drug plasma concentration). Our data are similar to what has been reported in adult patients and suggest that care taker report and medication return may overestimate the true level of adherence. Adherence to Antiretroviral therapy in paediatrics is critical in order to maximize the benefit of medication. Inadequate adherence is associated with immunological, and virological failure; drug resistance, and treatment failure [4]. In our study, high adherence levels to ART by caretaker report (98%) and medication return (97%) were observed. However, adherence level by nevirapine plasma concentration was significantly lower (85%) than the level estimated by caretaker report and medication return. Our findings are in agreement with those obtained in Cameroon in which a comparable proportion of patients were found to be adherent to ART by using nevirapine plasma concentration method [11].

In addition, there was a weak agreement in estimating adherence between nevirapine plasma concentration method and care taker report (kappa 0.09) as well as medication return (kappa 0.131). This means, non-adherent patients were classified as adherent by caretaker report and medication return methods whereas in fact, were non-adherent.

Using medication returns and care taker report, our data show that only 3% of the patients were non adherent to antiretroviral medication. These findings are similar those reported from Uganda where the level of adherence assessed using medication return and caretaker reporting was relatively high [20]. Nevirapine plasma concentration seems to give a better estimation of adherence to ART than caretaker report and medication return. Our study found a strong association between nevirapine plasma concentration (< 3 μg/ml) and immunosuppression. In addition, there was no association between medication return and immunosuppression. Similar findings have been reported from South Africa [13]. Furthermore, our study shows that caretaker report and medication return have low sensitivity to detect non adherent children compared to nevirapine plasma concentration, which could detect a higher proportion of non adherent children. At the moment, medication return and caretaker report, are methods used routinely for measuring adherence in paediatric patients on ART in HIV care and treatment clinics. This means that, a great deal of non-adherent patients receive suboptimal treatment and this has clinical implication in terms of treatment outcome and the prevention of the emergence and spread of drug resistance.

Our data show that nevirapine plasma concentration may be a better predictor of adherence and correlates well with the immunological response compared to medication return and caretaker report. However, lack of ability to detect missed dose through determination of drug concentration by HPLC, for drugs with a long half-life, such as nevirapine is one of the limitations of our study. Failure to detect missed doses would underestimate non adherence to ART in some patients. In addition, only a single blood sample was taken, and the time of last dosing was dependent on the authenticity of the caretaker’s reporting, which may not be guaranteed. It is possible that some of the caretakers or older children damped medication due to reasons previously reported [7] so as to create the impression of good adherence on the day of refill. Damping of pills to create false positive adherence has previously been reported [21].

In this study, non-adherence by nevirapine plasma concentration was found to be 15%, which is alarming when considering the management of HIV/AIDS in children**.** However, it is important to note that, blood drug concentration can be affected not only by the level of adherence but also drug bioavailability, individual’s metabolic capacity, timing of drug intake and the time the blood sample for determination of drug concentration is taken as well as having diarrhoea or vomiting immediately after drug administration. In our study, no patient was reported to have vomited or experienced diarrhoea after drug intake. It is unlikely that the low plasma concentrations observed in some patients have been due to poor nevirapine bioavailability since, in all patients, we used same paediatric fixed-dose combination tablets obtained from WHO prequalified manufacturers. In this study, all patients who were using other drugs, which could interact with nevirapine, such as ketoconazole and rifampicin were excluded precluding the possible influence of enzyme inhibition or induction on the observed plasma concentration. However, in this study, the patients were not genotyped with respect to CYP 3A and 2B6 to rule out the role of indivudual’s metabolic capacity that could influence the plasma concentrations of nevirapine. A recent Pharmacogenetics study conducted in Tanzanian adults indicated the frequencies of CYP2B6 genotypes to be 37%, 45% and 16% for homozygous wild type (fast metabolizers), heterozygous (intermediate) and homozygous mutated (poor metabolizers) repectively [22]. This implies that the obtained nevirapine plasma concentrations in some of our patients could have been influenced by the patient’s 2B6 activity. It is possible that observed high or low nevirapine concentrations were due to delay of blood sampling or drug intake, we recorded the last time of drug intake prior blood sampling and the time of blood sampling. We noted that the mean sampling interval was 4 ± 2 hours post intake of the last dose, which did not significantly deviate from the study protocol.

Despite the above limitations, our findings support the use of drug plasma concentration as an objective method for determining adherence to ART where feasible. This would complement the information obtained from care taker report and medication returns particularly in groups of patients showing poor treatment response. Determination of plasma drug concentration to estimate adherence is often not feasible but nevirapine plasma concentration may be useful and cost-effective in the setting of a pharmacovigilance program with adherence monitoring where this may be limited to patients at sentinel sites and at regular intervals. Apart from estimating adherence, nevirapine plasma concentration may be used in the analysis of non-response that may lead to detection of early signs of drug resistance particularly in patients with well characterised genotypes of important metabolizing enzymes.

## Conclusion

Lower level of adherence by nevirapine plasma concentration and its association with immunological response supports the validity of the method and indicates that the adherence estimate obtained by care takers’ reporting and medication returns may be an overestimate of true adherence level. This may have important clinical implications in antiretroviral therapy. Therefore, nevirapine plasma concentration may be a good predictor of adherence and should be considered where it is feasible and cost-effective.

## Competing interests

The authors declare that they have no competing interests.

## Authors’ contributions

MFW participated in the design of the study, coordination, data collection, data analysis and writing the manuscript. MO participated in the design, interpretation of nevirapine plasma concentration results, read and advised in writing final manuscript. MA participated in the design, advised and read the final manuscript. SP participated in design, data interpretation read and advised in writing the final manuscript. We have read and approved the final manuscript.

## Pre-publication history

The pre-publication history for this paper can be accessed here:

http://www.biomedcentral.com/1471-2431/13/95/prepub
